# Impact of Vasopressors on Microvascular Free Flap Perfusion in Head and Neck Reconstruction

**DOI:** 10.1002/micr.70095

**Published:** 2025-07-14

**Authors:** Mark Ooms, Philipp Winnand, Marius Heitzer, Nils Vohl, Marie Katz, Johannes Bickenbach, Frank Hölzle, Ali Modabber

**Affiliations:** ^1^ Department of Oral and Maxillofacial Surgery University Hospital RWTH Aachen Aachen Germany; ^2^ Department of Intensive Care Medicine University Hospital RWTH Aachen Aachen Germany

**Keywords:** flap perfusion, free flap, head and neck reconstruction, microsurgery, vasopressors

## Abstract

**Introduction:**

The use of vasopressors in microvascular head and neck reconstruction is still controversial in view of its potentially negative influence on microvascular flap perfusion, which is crucial for flap viability and commonly used as a parameter in flap monitoring. The aim of this study was to investigate the influence of vasopressors on microvascular free flap perfusion.

**Materials and Methods:**

Perfusion measurement data recorded intraoperatively and postoperatively using the Oxygen‐2‐see (O2C) analysis system in 274 patients undergoing microvascular head and neck reconstruction with fasciocutaneous free flaps (FFFs) or perforator free flaps (PFFs) between 2011 and 2020 were analyzed retrospectively. Vasopressor dose and perfusion parameters, such as flap blood flow, hemoglobin concentration, and hemoglobin oxygen saturation, as well as flap flow conductance (calculated as the ratio of flap blood flow and mean arterial blood pressure), were tested for associations.

**Results:**

Intraoperative hemoglobin oxygen saturation and postoperative flap blood flow were negatively associated with vasopressor dose in PFFs (*r* = −0.307, *p* < 0.001; *r* = −0.211, *p* = 0.012, respectively). Both associations remained in multivariable analysis (*p* = 0.002; *p* = 0.022, respectively). Postoperative flap flow conductance was negatively associated with vasopressor dose in PFFs (*r* = −0.232, *p* = 0.008). This association remained in multivariable analysis (*p* = 0.023).

**Conclusion:**

The use of vasopressors influences microvascular free flap perfusion in PFFs in terms of intraoperative hemoglobin oxygen saturation, postoperative flap blood flow, and postoperative flap flow conductance. This suggests that the use of vasopressors in PFFs may be an adjustable variable for controlling flap perfusion and should be considered a confounding variable during flap monitoring based on flap perfusion.

## 1 Introduction

Reconstruction in the head and neck region with microvascular free flaps is routinely performed, with superior functional and aesthetic results and generally low flap failure rates (Pattani et al. 2010; Abouyared et al. 2019; Shanker et al. 2021; Philteos et al. 2024). Nevertheless, the identification of risk factors to reduce microvascular free flap failure remains a current topic (Pattani et al. 2010; Philteos et al. 2024; Wax and Azzi 2018; Goh et al. 2019).

In particular, the use of vasopressors for intra‐ and postoperative hemodynamic management in microsurgical reconstructive procedures remains controversial because vasopressors may reduce microvascular free flap perfusion, which is a prerequisite for flap viability, and consequently contribute to flap failure (Philteos et al. 2024; Goh et al. 2019; Hölzle et al. 2006, 2010; Quinlan 2006; Miyamoto et al. 2008; Abdel‐Galil and Mitchell 2009; Ibrahim et al. 2014; McCauley et al. 2022; Burkhard et al. 2023; Safeek et al. 2023). The use of vasopressors in the context of microsurgical reconstructive procedures is the subject of two competing theories, with the first being that vasopressors reduce free flap perfusion due to the increased flap flow resistance resulting from the vasoconstriction of the flap vasculature and the second being that vasopressors promote free flap perfusion due to increased mean arterial blood pressure (Ibrahim et al. 2014; McCauley et al. 2022; Burkhard et al. 2023). Which theory is more appropriate remains unclear, as does the influence of vasopressors on microvascular free flap perfusion (Ibrahim et al. 2014; McCauley et al. 2022; Safeek et al. 2023).

Although several reviews have not found a negative impact on the part of vasopressors on microvascular free flap failure, the few studies addressing the influence of vasopressors on microvascular free flap perfusion were animal studies, included only a small number of patients, or did not evaluate the effect of commonly used vasopressors (e.g., norepinephrine) (Goh et al. 2019; Ibrahim et al. 2014; McCauley et al. 2022; Safeek et al. 2023; Eley et al. 2012; Swanson et al. 2016; Naik et al. 2020; Michelle et al. 2023; Noori et al. 2023). Knowledge about the influence of norepinephrine on microvascular free flap perfusion could help to improve both hemodynamic management in microvascular head and neck reconstruction and the monitoring of microvascular free flaps with the Oxygen‐2‐see (O2C) analysis system based on flap perfusion threshold values indicating vascular flap compromise (Wax and Azzi 2018; Hölzle et al. 2006, 2010; Burkhard et al. 2023; Hagau and Longrois 2009).

This study aimed to investigate the influence of norepinephrine on microvascular free flap perfusion in the head and neck region.

## 2 Material & Methods

### 2.1 Study Population

This retrospective study, based on routinely collected data, was conducted in accordance with the ethical standards of the institutional research committee and with the 1964 Helsinki Declaration and its later amendment, and was approved by the local ethics committee of the Medical Faculty RWTH Aachen University (EK 309–20).

The study included 274 patients who underwent microvascular reconstruction of the head and neck region after malignant or nonmalignant diseases with fasciocutaneous free flaps (FFFs; i.e., radial free forearm flaps) or perforator free flaps (PFFs; i.e., anterolateral thigh flaps or fibula free flaps) in our Department of Oral and Maxillofacial Surgery between 2011 and 2020. Incomplete datasets and age under 18 years were exclusion criteria.

Data on the baseline characteristics of the study population were collected from clinical records and surgery reports. Surgery duration was the time interval between the first incision and the last suture, and flap ischemia duration was the time interval between the cessation of flap perfusion at the donor site after pedicle transection and the reperfusion of the flap at the recipient site after the release of the anastomosis. Based on commonly used definitions, smoking was considered to be present if the patient currently smoked or had smoked daily for at least 6 months in the past (Latza et al. 2005). Prior neck dissection status was considered positive if an anatomic dissection of the recipient vessel in the form of a neck dissection had been performed, and prior irradiation status was considered positive if irradiation of the recipient vessel area in the form of neck irradiation had been performed prior to reconstructive surgery. Flap revision was defined as positive if a surgical anastomosis revision with return to the operating room was performed. Flap success was defined as negative if the flap was removed due to flap necrosis.

All surgical procedures were performed under general anesthesia. In terms of anastomoses, arterial anastomoses were created in an end‐to‐end manner, and the venous anastomoses were created in an end‐to‐side or end‐to‐end manner. All patients remained in the intensive care unit postoperatively, at least until the morning following surgery, with invasive mechanical ventilation, analgosedation, invasive arterial blood pressure measurement, and blood pressure regulation via central venous norepinephrine as required (target systolic blood pressure above 125 mmHg).

Flap conductance was calculated as the ratio of flap blood flow and mean arterial blood pressure (mean arterial blood pressure = diastolic blood pressure + 1/3 × [systolic blood pressure‐diastolic blood pressure]) (Eley et al. 2012; Whelton et al. 2018; Nakamura et al. 2020).

### 2.2 Flap Perfusion Measurement Data

The data for flap perfusion measurement were collected using the O2C analysis system (O2C Oxygen‐to‐see, LEA Medizintechnik, Giesen, Germany). Measurements were performed intraoperatively after the release of the anastomosis in the operating room and postoperatively on the first postoperative morning in the intensive care unit for 10 s (with a 4 s lead time) under ambient light compensation control at eight and two mm tissue depths, and the probe was placed centrally on the dried skin portion of the flap in a sterile sheath.

The O2C analysis system uses two measurement principles, namely Doppler spectroscopy (830 nm; 30 mW) to determine blood flow and white light spectroscopy (500–800 nm; 50 W) to determine hemoglobin concentration and hemoglobin oxygen saturation (Hölzle et al. 2006; Beckert et al. 2004). The measured values for blood flow (arbitrary units [AU]) were calculated by evaluating the Doppler shift of the laser light due to erythrocyte movement as a product of erythrocyte quantity and velocity (Hölzle et al. 2006; Beckert et al. 2004). The measured values for hemoglobin concentration (AU) and hemoglobin oxygen saturation (%) were calculated by evaluating the sum of white light absorbances and the white light color change in comparison to reference hemoglobin spectra with known oxygen saturation values, respectively (Hölzle et al. 2006; Beckert et al. 2004). The mean values of the measurements at eight and two mm tissue depths were used for further analysis.

### 2.3 Statistical Analysis

Patients were categorized according to their flap type (FFF or PFF) and American Society of Anesthesiologists score (ASA) (ASA > two or ASA ≤ two). The associations between intraoperative and postoperative vasopressor dose and flap flow conductance with flap perfusion parameters were analyzed separately for each flap type by calculating the Spearman correlation coefficient. Significant associations were analyzed using multiple regression analysis. For associations with vasopressor dose, adjustment was done for mean arterial blood pressure (mmHg), flap ischemia duration (min), and flap size (cm^2^) and for PFFs, additionally for flap type (anterolateral thigh flap vs. fibula free flap). For associations with flap conductance, adjustment was done for flap ischemia duration (min) and flap size (cm^2^) and for PFFs, additionally for flap type (anterolateral thigh flap vs. fibula free flap). Values of *p* < 0.05 were considered statistically significant. The statistical analysis was performed using SPSS Version 28 (SPSS, IBM, New York, USA).

## 3 Results

### 3.1 Clinical Characteristics of the Study Population

The study population included a total of 274 patients (146 patients reconstructed with FFFs and 128 patients reconstructed with PFFs [91 patients reconstructed with anterolateral thigh flaps and 37 patients reconstructed with fibula free flaps]) (Table 1). Flap revision was performed in eight FFFs and four PFFs due to venous congestion and in two PFFs due to arterial insufficiency.

**TABLE 1:** Clinical characteristics of the study population.

Variable	All (*n* = 274)	FFF (*n* = 146)	PFF (*n* = 128)
Sex *(n)*			
Male	140 (51.1%)	74 (50.7%)	66 (51.6%)
Female	134 (48.9%)	72 (49.3%)	62 (48.4%)
Age (years)	63.0 (18.0)	64.0 (17.0)	61.5 (21.0)
BMI (kg/m^2^)	24.3 (6.0)	24.9 (6.3)	23.6 (5.5)
ASA (*n*)			
1 + 2	154 (56.2%)	89 (61.0%)	65 (50.8%)
3 + 4	120 (43.8%)	57 (39.0%)	63 (49.2%)
Flap location *(n)*			
Tongue	42 (15.3%)	33 (22.6%)	9 (7.0%)
Floor of mouth	60 (21.9%)	38 (26.0%)	22 (17.2%)
Mandible	69 (25.2%)	15 (10.3%)	54 (42.2%)
Maxilla + Hard palate	37 (13.5%)	18 (12.3%)	19 (14.8%)
Cheek	23 (8.4%)	16 (11.0%)	7 (5.5%)
Soft palate	14 (5.1%)	11 (7.5%)	3 (2.3%)
Extraoral	29 (10.6%)	15 (10.3%)	14 (10.9%)
Arterial anastomosis recipient vessel *(n)*			
ECA	19 (6.9%)	4 (2.7%)	19 (11.7%)
FAA	104 (38.0%)	57 (39.0%)	47 (36.7%)
LIA	16 (5.8%)	4 (2.7%)	12 (9.4%)
STA	135 (49.3%)	81 (55.5%)	54 (42.2%)
Surgery duration (min)	545.0 (156.0)	514.0 (169.0)	561.0 (146.0)
Flap ischemia duration (min)	107.5 (35.0)	108.0 (34.0)	106.5 (40.0)
Diabetes *(n)*			
No	233 (85.0%)	124 (84.9%)	109 (85.2%)
Yes	41 (15.0%)	22 (15.1%)	19 (14.8%)
Arterial hypertension *(n)*			
No	180 (65.7%)	91 (62.3%)	89 (69.5%)
Yes	94 (34.3%)	55 (37.7%)	39 (30.5%)
Smoking status *(n)*			
No	168 (61.3%)	88 (60.3%)	80 (62.5%)
Yes	106 (38.7%)	58 (39.7%)	48 (37.5%)
Prior neck dissection *(n)*			
No	209 (76.3%)	122 (83.6%)	87 (68.0%)
Yes	65 (23.7%)	24 (16.4%)	41 (32.0%)
Prior neck irradiation *(n)*			
No	239 (87.2%)	135 (92.5%)	104 (81.3%)
Yes	35 (12.8%)	11 (7.5%)	24 (18.8%)
Flap survival *(n)*			
No	5 (1.8%)	2 (1.4%)	3 (2.3%)
Yes	269 (98.2%)	144 (98.6%)	125 (97.7%)
Flap revision *(n)*			
No	260 (94.9%)	138 (94.5%)	122 (95.3%)
Yes	14 (5.1%)	8 (5.5%)	6 (4.7%)

*Note:* Parameters are indicated as numbers (with percentage) for categorical data (sex, ASA, flap location, arterial anastomosis recipient vessel, diabetes, arterial hypertension, smoking status, prior neck dissection, prior neck irradiation, flap survival, flap revision) or median (with interquartile range) for metric data (age, BMI, surgery duration, flap ischemia duration) (separately described for all patients, patients reconstructed with a FFF, and patients reconstructed with a PFF).

Abbreviations: ASA, American Society of Anesthesiologists score; BMI, body mass index; ECA, external carotid artery; FAA, facial artery; FFF, fasciocutaneous free flap; LIA, lingual artery; PFF, perforator free flap; STA, superior thyroid artery.

### 3.2 Vasopressor Dose Values

The intraoperative median vasopressor doses related to intraoperative flap perfusion measurement in patients reconstructed with FFFs or PFFs were 0.092 μg/min per kg and 0.117 μg/min per kg, respectively (Table 2). The postoperative median vasopressor doses related to postoperative flap perfusion measurement in patients reconstructed with FFFs or PFFs were 0.105 μg/min per kg and 0.142 μg/min per kg, respectively.

**TABLE 2:** Vasopressor dose.

Variable	FFF (*n* = 146)	PFF (*n* = 128)
Intraoperative values		
Vasopressor (μg/min per kg)	0.092 (0.109)	0.117 (0.136)
Postoperative values		
Vasopressor (μg/min per kg)	0.105 (0.100)	0.142 (0.147)

*Note:* Parameters are indicated as median (with interquartile range) for vasopressor dose according to intraoperative values used as reference values for intraoperative flap perfusion measurement and postoperative values used as reference values for postoperative flap perfusion measurement (separately described for patients reconstructed with a FFF and patients reconstructed with a PFF).

Abbreviations: FFF, fasciocutaneous free flap; PFF, perforator free flap.

### 3.3 Associations Between Flap Perfusion Parameters and Vasopressor Dose

**FIGURE 1:**
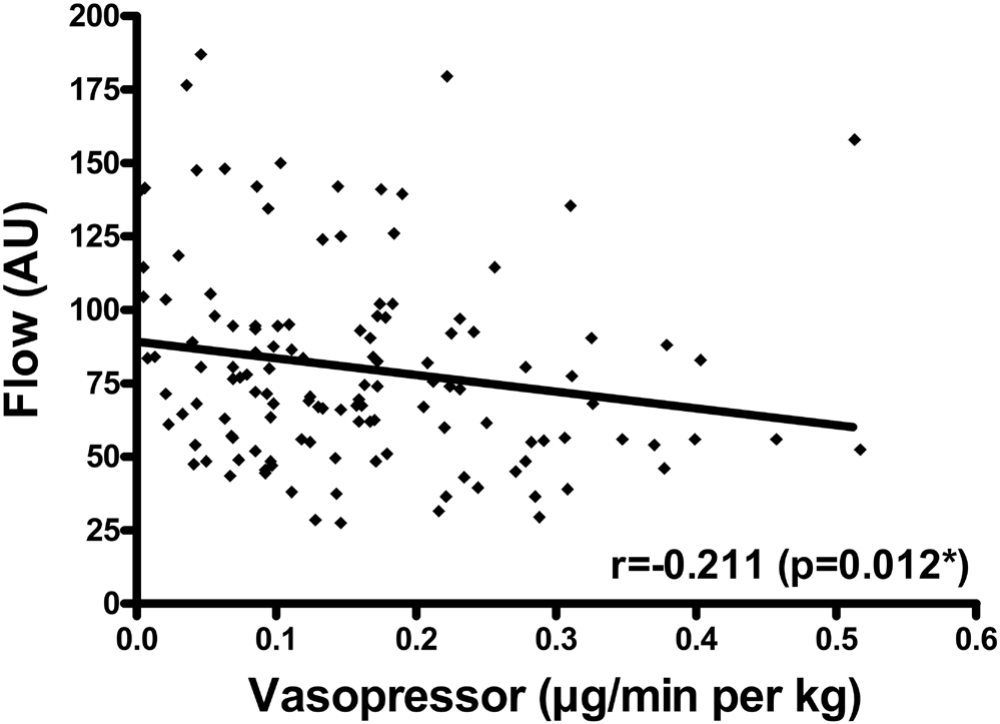
Postoperative flap blood flow and vasopressor dose in PFFs. Scatter plot for postoperative flap blood flow (AU) and vasopressor dose (μg/min per kg) in PFFs; *r* and *p* value corresponding to Spearman correlation coefficient (*p = 0.022 upon adjustment for mean arterial blood pressure (mmHg), flap ischemia duration (min), flap size (cm^2^), and flap type (anterolateral thigh flap vs. fibula free flap) in multiple regression analysis); abbreviations: AU, arbitrary units.

Intraoperative hemoglobin oxygen saturation and postoperative flap blood flow were negatively correlated with vasopressor dose in PFFs (*r* = −0.307, *p* < 0.001; *r* = −0.211, *p* = 0.012, respectively) (Table 3, Figure 1). Both associations persisted in multivariable testing (*p* = 0.002 and *p* = 0.022, respectively). No associations between flap perfusion parameters and vasopressor dose were observed in FFFs.

**TABLE 3:** Association between flap perfusion parameters and vasopressor dose.

Variable	FFF (*n* = 146)		PFF (*n* = 128)	
	*r*	*p*	*r*	*p*
Intraoperative measurement				
Flow (AU)	−0.018	0.830	−0.087	0.326
Hemoglobin concentration (AU)	0.102	0.223	0.109	0.222
Hemoglobin oxygen saturation (%)	−0.094	0.259	**−0.307**	< 0.001*
Postoperative measurement				
Flow (AU)	0.009	0.909	**−0.211**	0.012*
Hemoglobin concentration (AU)	0.111	0.183	0.062	0.485
Hemoglobin oxygen saturation (%)	0.113	0.176	−0.048	0.590

*Note:* Parameters are indicated as Spearman correlation coefficient (*r*) with *p* value (separately described for patients reconstructed with a FFF and patients reconstructed with a PFF); significant *p* values are bold (*p < 0.05 upon adjustment for mean arterial blood pressure (mmHg), flap ischemia duration (min), flap size (cm^2^), and flap type (anterolateral thigh flap vs. fibula free flap) in multiple regression analysis).

Abbreviations: AU, Arbitrary units; FFF, fasciocutaneous free flap; PFF, perforator free flap.

### 3.4 Flap Flow Conductance Values

The intraoperative flap flow conductance values in patients reconstructed with FFFs or PFFs were 1.017 and 0.862 AU/mmHg, respectively (Table 4). The postoperative flap flow conductance values in patients reconstructed with FFFs or PFFs were 1.020 and 0.916 AU/mmHg, respectively.

**TABLE 4:** Flap flow conductance.

Variable	FFF (*n* = 146)	PFF (*n* = 128)
Intraoperative values		
Flow conductance (AU/mmHg)	1.017 (0.683)	0.862 (0.585)
Postoperative values		
Flow conductance (AU/mmHg)	1.020 (0.678)	0.916 (0.474)

*Note:* Parameters are indicated as median (with interquartile range) for flap flow conductance according to intraoperative values and postoperative values (separately described for patients reconstructed with a FFF and patients reconstructed with a PFF).

Abbreviations: AU, Arbitrary units; FFF, fasciocutaneous free flap; PFF, perforator free flap.

### 3.5 Association Between Flap Flow Conductance and Vasopressor Dose

**FIGURE 2:**
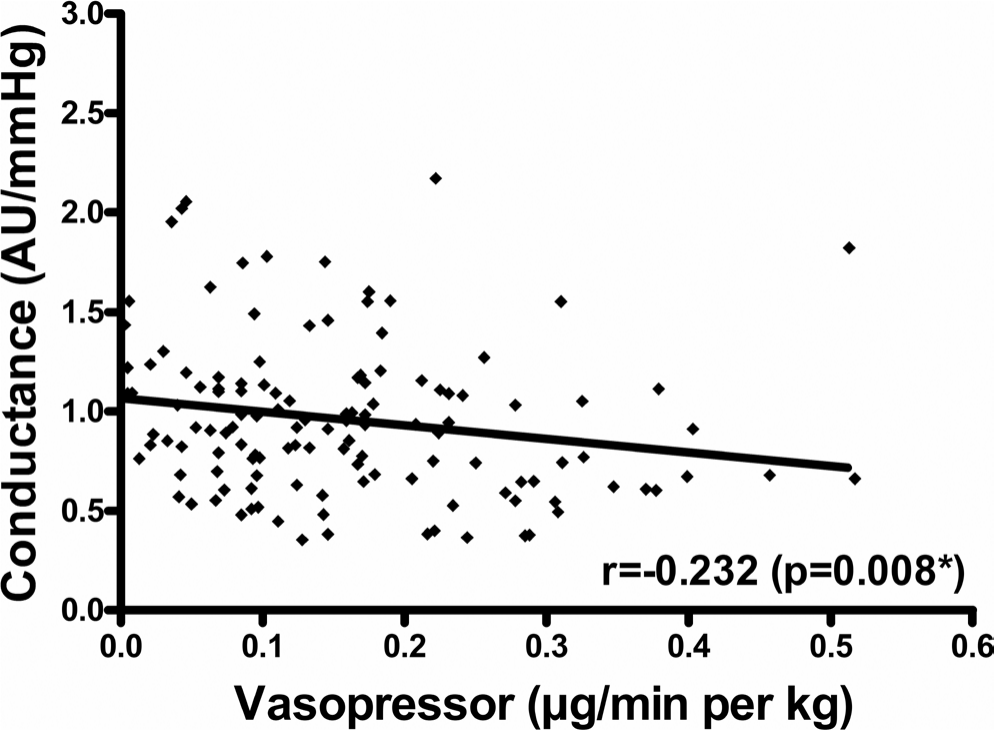
Postoperative flap flow conductance and vasopressor dose in PFFs. Scatter plot for postoperative flap flow conductance (AU/mmHg) and vasopressor dose (μg/min per kg) in PFFs; *r* and *p* value corresponding to Spearman correlation coefficient (*p = 0.023 upon adjustment for flap ischemia duration (min), flap size (cm^2^), and flap type (anterolateral thigh flap vs. fibula free flap) in multiple regression analysis); abbreviations: AU, arbitrary units.

Postoperative flap flow conductance was negatively correlated with vasopressor dose in PFFs (*r* = −0.232, *p* = 0.008) (Table 5, Figure 2). This association persisted in multivariable testing (*p* = 0.023). No association between flap flow conductance and vasopressor dose was observed in FFFs.

**TABLE 5:** Association between flap flow conductance and vasopressor dose.

Variable	FFF (*n* = 146)		PFF (*n* = 128)	
	*r*	*p*	*r*	*p*
Intraoperative values				
Flow conductance (AU/mmHg)	−0.007	0.931	−0.109	0.220
Postoperative values				
Flow conductance (AU/mmHg)	0.000	1.000	**−0.232**	0.008*

*Note:* Parameters are indicated as Spearman correlation coefficient (*r*) with *p* value (separately described for patients reconstructed with a FFF and patients reconstructed with a PFF); significant *p* values are bold (*p < 0.05 upon adjustment for flap ischemia duration (min), flap size (cm^2^), and flap type (anterolateral thigh flap vs. fibula free flap) in multiple regression analysis).

Abbreviations: AU, Arbitrary units; FFF, fasciocutaneous free flap; PFF, perforator free flap.

## 4 Discussion

This study investigated the influence of vasopressors on microvascular free flap perfusion, as the use of vasopressors for intra‐ and postoperative hemodynamic management in microsurgical reconstructive procedures remains controversial due to the suspected impairment of flap perfusion and the resulting risk of flap failure (Philteos et al. 2024; Goh et al. 2019; Hölzle et al. 2006, 2010; Quinlan 2006; Miyamoto et al. 2008; Abdel‐Galil and Mitchell 2009; Ibrahim et al. 2014; McCauley et al. 2022; Burkhard et al. 2023; Safeek et al. 2023).

While it has been shown that the use of vasopressors in microsurgical reconstructive procedures is generally not associated with a higher risk of flap failure, studies investigating the influence of vasopressors on microvascular flap perfusion are scarce, and those regarding commonly used FFFs (i.e., radial free forearm flaps) and PFFs (i.e., anterolateral thigh flaps and fibula free flaps) as well as vasopressors (i.e., norepinephrine) are lacking (Goh et al. 2019; Hölzle et al. 2010; Ibrahim et al. 2014; McCauley et al. 2022; Safeek et al. 2023; Eley et al. 2012; Swanson et al. 2016; Naik et al. 2020; Michelle et al. 2023; Noori et al. 2023). The knowledge gained via additional studies could improve both intra‐ and postoperative hemodynamic management and help balance general circulation and regional flap perfusion, and flap monitoring with the O2C analysis system which is based on predefined threshold values for flap perfusion parameters and used for clinical decision making regarding flap revision (Abouyared et al. 2019; Wax and Azzi 2018; Hölzle et al. 2006, 2010; Quinlan 2006; McCauley et al. 2022; Burkhard et al. 2023; Hagau and Longrois 2009).

In this study, microvascular free flaps were analyzed separately for FFFs and PFFs, as these flap types differ in their vascular anatomies and the flap perfusion parameter values measured with the O2C analysis system (Hölzle et al. 2006, 2010; Coscia and Rubino 2005; Rubino et al. 2006; Yu 2014; Sham et al. 2018). In addition to the influence of vasopressors on flap perfusion parameters, the influence of vasopressors on flap flow conductance was also evaluated to account for the influence of mean arterial blood pressure (Whelton et al. 2018; Nakamura et al. 2020).

This study found that flap perfusion in PFFs was influenced by norepinephrine dose in terms of a negative association with postoperative flap blood flow and flap flow conductance.

These findings are consistent with the results of another study investigating the initial effect of vasopressors (e.g., norepinephrine) on flap perfusion after surgery in patients undergoing microvascular head and neck reconstruction, which showed that norepinephrine decreased flap flow conductance (Eley et al. 2012). In contrast to the results of the present study, that other study also found that norepinephrine increased flap blood flow, though no adjustment was made for blood pressure. However, the different timings of flap perfusion measurement, the small number of patients, and the lack of separate analyses for FFFs and PFFs make it difficult to compare the results between the studies. The findings of this study, in terms of a negative association between norepinephrine dose and flap blood flow in PFFs, were expected given a theoretical model suggesting that the blood pressure gradient, specifically mean arterial blood pressure, vessel radius, vessel length, and blood viscosity, are determinants of tissue perfusion, and thus flap perfusion (Wax and Azzi 2018; Quinlan 2006; McCauley et al. 2022). Norepinephrine increases blood pressure and decreases vessel radius in terms of vasoconstriction, theoretically leading to an overall decrease in flap perfusion and flap flow conductance, as blood pressure has an effect at the first power but vessel radius has an effect at the fourth power (Wax and Azzi 2018; Quinlan 2006; McCauley et al. 2022; Al Saied et al. 2020). In general, the presence of this association only in PFFs could be due to the vascular anatomy of PFFs, which is based on serial flow resistances, with few perforator vessels and a decreasing total vessel diameter from the flap pedicle to the microcirculation, making PFFs presumably more susceptible to the vasoconstrictive effect of norepinephrine than FFFs, which are based on parallel flow resistances, with multiple perforator vessels and an increasing vessel diameter from the flap pedicle to the microcirculation (Coscia and Rubino 2005; Rubino et al. 2006; Yu 2014; Sham et al. 2018; Weyh and Fernandes 2021). Furthermore, the presence of this association only postoperatively could be related to a delayed hypersensitivity of the flap tissue to norepinephrine due to the loss of modulating autonomic input, as the sympathetic input to the flap tissue was dissected at the donor site (Eley et al. 2012; Al Saied et al. 2020).

The negative associations observed between intraoperative hemoglobin oxygen saturation and norepinephrine in PFFs, despite there being no association between flap blood flow and norepinephrine, are unexpected, as hemoglobin oxygen saturation is generally positively associated with flap blood flow in the form of flap perfusion as measured with the O2C analysis system (Hölzle et al. 2010).

Overall, the observed associations were all quantitatively weak, but they persisted in multivariable analysis after adjustment for potentially confounding variables (Schober et al. 2018).

The limitations of this study are that flap perfusion was only measured at one central point of the flap, capturing only a small portion of the flap microcirculation, and only at two time points, representing only a short period of time after flap reperfusion (Miyamoto et al. 2008; Eley et al. 2012; Dusseldorp and Pennington 2014). However, data for potentially confounding variables, such as blood pressure, were only available for the two measurement time points used in this study, and flap perfusion measurement in the context of flap monitoring is often performed at one central point on the flap (Hölzle et al. 2006, 2010; de Backer and Foulon 2019). In addition, other potentially confounding variables related to flap perfusion (e.g., vessel length and the diameter of the cervical recipient vessels or flap pedicle vessels, flow of the cervical recipient vessels, flap thickness, or technical aspects of the microanastomosis) cannot be excluded. In general, norepinephrine doses were not extremely low or high, which may limit the applicability of the study results, and individual responses of patients to vasopressors (e.g., due to long term vasopressor dependency in previous surgeries) were not taken into account.

This exploratory study provides insights into microvascular free flap perfusion and demonstrates that flap perfusion is partially dependent on norepinephrine dose, particularly flap blood flow in PFFs. In terms of clinical implications, this emphasizes that in PFFs, norepinephrine dose may serve as a parameter for use in controlling flap perfusion and that decreasing norepinephrine dose, with consequent increases in flap perfusion, may contribute to flap survival because decreased flap perfusion has been linked to flap failure (Hölzle et al. 2006, 2010; Quinlan 2006; Miyamoto et al. 2008; Abdel‐Galil and Mitchell 2009; McCauley et al. 2022; Burkhard et al. 2023). In this context, it should be mentioned that the evidence for the lack of an association between vasopressors and flap failure in microvascular reconstructive procedures, with a focus on head and neck reconstruction, is based on studies that had few patients, did not differentiate between FFFs and PFFs, or did not focus on norepinephrine, which in combination with a generally low flap failure rate makes it difficult to draw conclusions (Naik et al. 2020; Michelle et al. 2023; Raittinen et al. 2016; Rose et al. 2016; Rajan et al. 2019). Thus, the choice of PFFs for microvascular head and neck reconstruction in patients for whom difficult intra‐ and postoperative hemodynamic management, especially the need for high vasopressor doses, is expected (e.g., patients with cardiovascular comorbidities) should be critically evaluated (Abouyared et al. 2019; McCauley et al. 2022; Burkhard et al. 2023; Naik et al. 2020; Brinkman et al. 2013). Also, in cases with an absolute need for PFFs in the form of anterolateral thigh flaps due to the need for a large amount of tissue or fibula free flaps due to the need for bone reconstruction, vasopressors with a less vasoconstrictive effect, especially in terms of the reduction of the vessel radius in the flap tissue (e.g., epinephrine) may be beneficial (McCauley et al. 2022; Naik et al. 2020; Weyh and Fernandes 2021; Wei et al. 2002). In addition, in flap monitoring based on flap perfusion measurement with the O2C analysis system, norepinephrine dose should be considered a confounding variable in the context of predefined absolute threshold values, such as that for flap blood flow (Hölzle et al. 2006, 2010). Further studies are needed to confirm these results, and prospective studies that evaluate all variables potentially affecting flap perfusion and measure flap perfusion multiple times (before flap harvest, after flap harvest, after anastomosis, and before flap inset) could add valuable information on the impact of vasopressors on microvascular free flap perfusion.

## 5 Conclusion

The results of this study indicate that microvascular free flap perfusion in PFFs is influenced by norepinephrine dose, as postoperative flap blood flow in PFFs was negatively associated with norepinephrine dose. This emphasizes that norepinephrine dose can be used to control flap perfusion in PFFs, which could help ensure flap viability and survival. This also implies that norepinephrine dose should be considered a confounding variable in the context of flap monitoring with the O2C analysis system with respect to absolute threshold values indicating vascular flap compromise, such as that for flap blood flow.

## Ethics Statement

Study design and methodology were reviewed and approved by the local ethics committee of the Medical Faculty of RWTH Aachen University (EK 309–20). The local ethics committee of the Medical Faculty of RWTH Aachen University allowed us to waive informed consent for this human study. All methods were in accordance with the relevant guidelines and regulations.

## Conflicts of Interest

The authors declare no conflicts of interest.

## Data Availability

The data that support the findings of this study are available from the corresponding author upon reasonable request.
